# Nutrient digestibility, rumen parameters, and (cannabinoid) residues in sheep fed a pelleted diet containing green hemp (*Cannabis sativa* L.) biomass

**DOI:** 10.1093/tas/txac141

**Published:** 2022-10-26

**Authors:** S A Stevens, G L Krebs, C J Scrivener, G K Noble, B L Blake, K C Dods, C D May, Z X Tai, E H Clayton, E E Lynch, K N Johnson

**Affiliations:** School of Agricultural, Environmental and Veterinary Sciences, Charles Sturt University, Wagga Wagga, NSW 2678, Australia; School of Agricultural, Environmental and Veterinary Sciences, Charles Sturt University, Wagga Wagga, NSW 2678, Australia; Noble Animal Health Consultancy, Wagga Wagga, NSW 2678, Australia; School of Agricultural, Environmental and Veterinary Sciences, Charles Sturt University, Wagga Wagga, NSW 2678, Australia; Department of Primary Industries and Regional Development, Bunbury, WA 6230, Australia; ChemCentre, Bentley, WA 6983, Australia; ChemCentre, Bentley, WA 6983, Australia; ChemCentre, Bentley, WA 6983, Australia; NSW Department of Primary Industries, Wagga Wagga Agricultural Institute, Wagga Wagga, NSW 2650, Australia; School of Agricultural, Environmental and Veterinary Sciences, Charles Sturt University, Wagga Wagga, NSW 2678, Australia; School of Agricultural, Environmental and Veterinary Sciences, Charles Sturt University, Wagga Wagga, NSW 2678, Australia

**Keywords:** hemp, novel forage, summer crops, THC

## Abstract

The feeding value for ruminants of green hemp biomass, from the low Δ^9^-tetrahydrocannabinol (Δ^9^-THC) variety of *Cannabis sativa* L., is unknown. Twelve Merino ewes were individually penned and randomly allocated on a stratified liveweight basis to one of two pelleted dietary treatments, control (0% hemp, *n* = 6) or hemp (42% green hemp biomass, *n* = 6) that delivered a diet meeting the nutrient requirements of the animals. The experimental period consisted of 17 d dietary and housing adaptation, followed by 7 d total urine and feces collection for determination of apparent nutrient digestibility. A ruminal fluid sample was collected on day 27 and assessed for pH, ammonia, volatile fatty acid (VFA), and cannabinoid concentrations. A blood sample from the jugular vein and incisional subcutaneous fat biopsy from an area around the base of the tail were collected on day 28 with additional fat biopsies taken 35 d and 140 d post-feeding to measure cannabinoids. The dry matter (DM), organic matter (OM), and crude protein (CP) digestibilities, along with total VFA concentration did not differ (*P* = 0.713) between the two diets; however, acid detergent fiber (ADF) and neutral detergent fiber (NDF) digestibilities (*P* < 0.001), water intake (*P* = 0.023), and fecal water output (*P* < 0.001) were significantly lower for the sheep-fed Hemp. Rumen pH did not vary (*P* = 0.256) between diets, but ruminal ammonia concentration was significantly lower (*P* = 0.024) for sheep consuming Hemp. Sheep-fed Hemp had significantly greater molar proportions of butyric (*P* = 0.039) and hexanoic (*P* = 0.012) acids and lower molar proportions of propionic acid (*P* = 0.003). There were no differences between diets for N intake (*P* = 0.175), fecal N output (*P* = 0.253), and N balance (*P* = 0.695), with all sheep in positive N balance; however, there was significantly lower (*P* = 0.001) urinary N output for sheep-fed Hemp. Cannabidiolic acid (CBDA) and tetrahydrocannabinolic acid (THCA) were detected in plasma of all sheep-fed Hemp. ∆^9^-tetrahydrocannabinol was present in the subcutaneous fat of four of the six sheep on the final day of being fed Hemp, and in all (six) sheep 35 d post-feeding and one sheep 140 d post-feeding Hemp. No cannabinoids were detected in the corresponding samples taken from the sheep-fed Control. Thus, despite green hemp biomass being nutritionally a suitable feed for ruminants, under current Food Standards in Australia, the presence of these cannabinoid residues restricts its use in ruminant diets.

## INTRODUCTION

Hemp is the low Δ^9^-THC variety of *Cannabis sativa* L. and has been identified as a potential summer forage option for ruminants (Agrifutures, 2020) due to its fast growth rate and high biomass production. Very few studies have investigated the value and safety of hemp forage as a livestock feed. In most countries it cannot legally be fed to livestock over concerns Δ^9^-THC will accumulate in animal products destined for the human food market (EFSA, 2011). Currently there is no safe or “maximum level” set by Food Standards Australia and New Zealand (FSANZ). Therefore, in Australia, the tolerance for Δ^9^-THC in products of animal origin (meat, milk, eggs) is zero (FSANZ, 2012).

In terms of feeding value, Krebs et al. (2021) demonstrated inclusion of up to 56% hemp stubble had no effect on DM intake, live weight gain, or the feed-to-gain ratio but positively impacted nutrient digestibility in sheep. However, cannabinoids were detected in the liver and subcutaneous fat after being fed for 56 d (Krebs et al., 2021). Cannabinoids have a high affinity to adipose tissue due to being lipophilic, leading to storage in animal tissues (Kleinhenz et al., 2020b).

With feeding value and cannabinoid residues in mind, the aim of this study was to investigate nutrient digestibility, rumen parameters, water intake, nitrogen (N) balance and cannabinoid residues for sheep fed a pelleted diet containing green hemp biomass.

## MATERIALS AND METHODS

The study was conducted at the Department of Primary Industry (DPI) Animal Nutrition Unit at Wagga Wagga, NSW. The use and care of animals for the project were approved by Charles Sturt University’s (CSU) Animal Care and Ethics Committee (Protocol number: A21096) and by the DPI Animal Care and Ethics Committee (Protocol number: ORA 21/24/014C). The study was compliant with the Animal Research Act 1985 (as amended) in accordance with the Australian Code of Practice for the Care and Use of Animals for Scientific Purposes.

### Experimental Animals and Housing

Twelve Merino ewes (45.2 ± 1.40 kg; mean ± SE) were housed indoors, in either individual pens (1.2 × 2.4 m) or metabolism cages (1.0 m × 1.5 m). The metabolism cages enabled the daily collection of feces and urine during the digestibility study. Once the digestibility study was completed, the ewes were returned to their individual pens. Sheep had access to fresh, clean water and were able to view each other throughout the entire experimental period. At the conclusion of the feeding trial, the sheep-fed Hemp was released into a nearby paddock to allow for subsequent collection of (post-feeding) subcutaneous fat biopsies to determine cannabinoid residues.

### Experimental Design

The experimental period totaled 28 d including a 17 d dietary and housing adaptation and a 7 d digestibility study. Two experimental diets with six replicates per diet were tested. The sheep were randomly allocated on a stratified liveweight basis to the two dietary treatments and then randomly allocated to individual pens.

### Experimental Diets

The diets were formulated, pelleted diets that contained either 0% (Control) or 42% green hemp biomass (Hemp). The hemp crop, a mix of the varieties YUMA and HanNE, was grown at the Department of Primary Industries and Regional Development Research Station at Kununurra, WA. At harvest, HanNE (approx. 30% crop) was in full flower/early seed while YUMA was in early flower. This harvest time was selected to represent a crop peaking in cannabinoid content as a worst-case scenario with regard to the cannabinoid intake of animals consuming such a crop. The crop was allowed to wilt before baling and then chopped using an industrial chaff cutter. The hemp hay contained 8.2 g CP/100 g DM, 42 g ADF/100 g DM, 52 g NDF/100 g DM, 0.001% cannabichromene (CBC), 0.001% cannabigerol (CBG), 0.014% cannabigerolic acid (CBGA), 0.001% tetrahydrocannabivarin (THCV), 0.079% tetrahydrocannabinolic acid (THCA), 0.008% ∆^9^-THC, 0.192% cannabidiolic acid (CBDA), 0.010% cannabidiol (CBD), 0.077% total THC, and 0.177% total CBD (all cannabinoids were analyzed on w/w basis). Total CBD was calculated by CBD + (0.877 * CBDA); total THC was calculated based upon Δ^9^-THC + (0.877 * THCA).

The two pelleted diets were formulated to be a complete diet as well as isocaloric and isonitrogenous (Specialty Feeds, Glen Forrest, WA). Due to the limited supply of hemp hay, only a 42% inclusion level could be used for the Hemp dietary treatment. The nutritive value of the hemp hay differed from that of the barley straw (used as the alternative fiber source in Control); therefore, to achieve isocaloric and isonitrogenous pellets there was a difference in the ingredient composition of the experimental diets (Table 1). The nutritive value and cannabinoid content of the diets are presented in Table 2.

**Table 1.** Composition of the experimental diets, 0% (control) and 42% hemp biomass (hemp)

**Ingredient**	**Inclusion level, %**	
	**Control**	Hemp
Barley	29.202	49.770
Oats: milling	10.001	–
Lupins: whole	17.391	2.666
Barley straw	37.153	–
Hemp hay (pre-chaffed)	–	42.000
Salt	0.196	0.268
Gypsum	1.000	1.000
Dicalcium phosphate	2.488	–
Industrial lime	0.500	0.500
EMag	0.141	–
Potassium chloride	1.022	–
Monoammonium phosphate	0.081	1.659
Lysine	–	0.114
Methionine	–	0.073
Premix (micro)	0.200	0.200
Canola oil	0.625	1.751

**Table 2.** Nutritional and cannabinoids profiles of the experimental diets, 0% (control) and 42% hemp biomass (hemp)

**Parameter**	**Control**	**Hemp**
Dry matter, g/kg as fed	0.93	0.93
Metabolizable energy, MJ/kg DM	10.6	10.9
Crude protein, g/100 g DM	11.3	10.6
Organic matter, g/100 g DM	93.71	91.83
Neutral detergent fiber, g/100 g DM	38.0	30.5
Acid detergent fiber, g/100 g DM	21.3	18.2
Starch, g/100 g DM	22.44	33.30
Fat, g/100 g DM	3.9	3.7
Polyphenolics, mg/kg DM	1890	3130
Calcium, g/kg DM	13	11
Cobalt, mg/kg DM	0.73	1.10
Copper, mg/kg DM	10.0	9.3
Iron, mg/kg	280	1100
Magnesium, g/kg DM	17	22
Phosphorus, g/kg DM	73	67
Potassium, g/kg DM	64	84
Sodium, g/kg DM	14	15
Zinc, mg/kg DM	86	67
Cannabichromene (CBC), % w/w	<0.001	<0.001
Cannabidiol (CBD), % w/w	<0.001	0.005
Cannabidiolic acid (CBDA), % w/w	<0.001	0.041
Cannabidivarin (CBDV), % w/w	<0.001	<0.001
Cannabigerol (CBG), % w/w	<0.001	<0.001
Cannabigerolic acid (CBGA), % w/w	<0.001	0.002
Cannabinol (CBN), % w/w	<0.001	< 0.001
Δ^8^-tetrahydrocannabinol (Δ^8^-THC), % w/w	<0.001	<0.001
Δ^9^-tetrahydrocannabinol (Δ^9^-THC), % w/w	<0.001	0.003
Tetrahydrocannabinolic acid (THCA), % w/w	<0.001	0.014
Tetrahydrocannabivarin (THCV), % w/w	<0.001	<0.001
Total cannabidiol (CBD), % w/w	<0.001	0.040
Total tetrahydrocannabinol (THC), %	<0.001	0.015

Metabolizable energy calculated using the formula: ME = 0.164 × (%DMD + %Fat) − 1.6 (Oddy et al., 1983).

Dry matter digestibility calculated using the formula: DMD = 83.58 − (NDF × 0.824) + (protein × 0.42) (Oddy et al., 1983).

<signifies a result is less than the limit of quantitation for the method.

Total THC was calculated based upon THC + 0.877 * THCA.

Total CBD was calculated based upon CBD + 0.877 * CBDA.

After a 2-wk dietary adaptation phase, the sheep were transferred to the metabolism cages for a 3 d adaptation period followed by the 7 d digestibility study.

The sheep were fed ad libitum twice daily at 0900 and 1600 hours. The quantity of feed offered, and orts were recorded daily for the entire experimental period. Daily water intake was also recorded during the 7 d digestibility study.

### Digestibility Study

For the duration of the digestibility study, the orts (from the previous day) were collected prior to feeding. The orts were weighed, recorded, and retained for subsequent analysis of nutrient content. Urine was collected into a bucket containing 100 mL of 10% H_2_SO_4_. After recording the amount excreted each day, a 10% subsample (of urine) was taken and stored at −18 °C pending chemical analysis. The weight of feces voided daily was recorded, and after thorough mixing, a 10% subsample was taken and stored at −18 °C for subsequent chemical analysis.

### Ruminal Fluid Samples

On day 27 (3 d after completion of the digestibility study), a ruminal fluid sample was collected from each sheep approximately 3 h after the morning feed using a stomach tube. Each sample was assessed for saliva contamination, and if contaminated the sample was discarded and a subsequent sample was taken. If the second sample was also contaminated, the sample was kept, but the value was not included in the analysis for ruminal fluid pH (Packer et al., 2011). The ruminal fluid samples were collected from the sheep-fed Control and then from the sheep-fed Hemp to avoid possible cross-contamination with cannabinoids.

Immediately after collection of the sample (approximately 100 mL), the pH of the ruminal fluid was measured and recorded. Three subsamples of approximately 20 mL of ruminal fluid were immediately stored at −18 °C for subsequent analysis of ruminal ammonia (NH_3_), VFA, and cannabinoid concentrations. The subsample for NH_3_ analysis was acidified with 0.5 mL H_2_SO_4_ (the pH of the samples was checked to ensure they were highly acidic).

### Fat Biopsy and Blood Collection

On the final day of the feeding period (i.e., 22 d of full feeding of the experimental diets) and then again 35 d and 140 d post-feeding the experimental diets, a sample of subcutaneous fat was collected from each sheep via an incisional biopsy by a registered veterinarian. Wool was removed from an area around the base of the tail and the area was anesthetized with 2 mL lignocaine (Nopaine 2%). A sample (approximately 5 mm diameter) of subcutaneous fat was collected via a 1 cm incision (Finch et al., 2012). The skin was then closed with two interrupted sutures. The fat sample was stored at −18 °C for analysis of cannabinoids.

On the final day of the feeding period and immediately after collection of the fat sample, a blood sample was collected from the jugular vein of each sheep using a heparinized vacutainer and an 18-gauge needle and immediately placed on ice. The blood samples were then centrifuged at 3,000 rpm for 10 min at 4 °C and the plasma was decanted and stored at −18 °CC until analysis for cannabinoid concentrations.

### Sample Preparation

As fed and orts collected during the digestibility study were bulked for each sheep for subsequent analysis of chemical composition (ash, CP, NDF, and ADF).

The frozen fecal samples were thawed at room temperature and combined to create a composite sample for each sheep. The composite samples were dried at 80 °C to constant weight to determine the DM content. A subsample of the dried composite samples (approx. 100 × *g*) was used for analysis of ash, CP, NDF, and ADF contents.

The frozen urine samples were thawed at room temperature and combined to form a composite sample for each sheep. A subsample (100 mL) was taken for each sheep, then stored at −18 °C for subsequent analysis of N content.

### Analytical Procedures

ChemCentre conducted all analytical procedures except for the VFA analysis which was undertaken at the NSW DPI Feed Laboratory at Wagga Wagga, NSW.

Analytical dry matter and ash content of the feed, orts, and fecal samples were analyzed according to the procedure of AOAC (2000) and AOAC (2000), respectively. The Dumas combustion method using a Leco CNS 2000 analyzer (Leco, St Joseph, MI, USA) was used to determine the N content of feed, orts, and feces (0.2 g ground dry sample). The CP content of the samples was determined by multiplying the N content by the factor of 6.25. The ADF and NDF contents of the ground feed, orts, and feces samples (AFIA, 2014) were determined using the ANKOM Fiber Analyzer (Ankom 200/220 fiber analyzer, ANKOM technology, Macedon NY, USA).

Following thawing and centrifugation at 1,500 × *g* for 10 min, the NH_3_-N concentration of the ruminal fluid samples was determined using flow injection analyzer spectroscopy (Lachat 8000 series FIA). This method was adapted from Lachat (2012), with ammonia chloride as the standard.

Volatile fatty acid concentrations and molar proportions of the ruminal fluid samples were as previously described by Krebs et al. (2021).

Cannabinoids and associated metabolites in Hemp diet, subcutaneous fat, plasma, and ruminal fluid samples were determined using LC−MS/MS as described by Krebs et al. (2021). The subcutaneous fat samples were freeze-dried prior to extraction; the plasma samples were analyzed as received; and the ruminal fluid samples were centrifuged (3,000 × *g* for 5 min) prior to analysis. An aliquot of the sample, spiked with deuterated internal standards, was extracted in methanol prior to matrix clean-up using solid phase extraction (SPE). Samples were analyzed using an Agilent 6470A LC-MS/MS and quantified against certified reference standards (Cerilliant, USA). The limits of detection and quantification are presented in Table 3.

**Table 3.** Limits of detection (LoD) and quantification (LoQ) of cannabinoids and metabolites in plasma and fat (Krebs et al., 2021)

**Compound**	**Fat**		Plasma	
	LoD	LoQ	LoD	LoQ
	**(μg/kg DM)**	**(μg/kg DM)**	**(μg/L)**	**(μg/L)**
∆ ^9^-THC	<15	50	<2	5
THCA	<30	100	<2	5
CBD	<15	50	<2	5
CBDA	<15	50	<2	5
11-nor-9-carboxy-∆ ^9^-tetrahydrocannabinol	<15	50	<2	5
11-hydroxy-∆ ^9^-tetrahydrocannabinol	<15	50	<2	5
11-nor-9-carboxy-∆^ 9^-tetrahydrocannabinol glucuronide	<15	50	<2	5

### Calculations

#### Apparent nutrient digestibility.

Nutrient intake for each animal was calculated by subtracting the amount of each constituent (DM, OM, CP, NDF, and ADF) in the feed refusals from that in the feed offered. On a DM basis, the digestibility of the nutrients (DM, OM, CP, NDF, and ADF) was calculated using the following formula (AFIA, 2014):

Digestibility(%)=[(dietary intakefaecal output)/dietary intake] x 100

#### Molar proportions and ratios of volatile fatty acids concentrations.

The molar proportions of individual concentrations were calculated using the total VFA and individual VFA concentration. Using concentrations (or molar proportions) of individual VFA, the ratios of acetic acid to propionic acid (Ac:Pr) and the propionic acid to acetic acid plus two times butyric acid (Pr: Ac + 2 × Bu) were calculated to assist in explaining the energy partitioning of ATP between the individual VFA.

#### Nitrogen balance.

Nitrogen balance was determined for the 7 d collection period and calculated using the following equation:


$$\begin{array}{l} N\;balance\;\left(g/d\right)=\;average\;daily\;N\;intake\;\left(g/d\right)\\ (average\;daily\;faecal\;N\;output\;\left(g/d\right)\\ +\;average\;daily\;urinary\;N\;output\;\left(g/d\right)) \end{array}$$

Average daily N intake was calculated by multiplying the average daily DM intake by the N content of the feed; average daily fecal N output was calculated by multiplying the average daily fecal DM weight by the N content of the feces and the average daily urinary N was calculated by multiplying the average daily urinary output by the N content of the urine.

### Statistical Analysis

Before analysis, all data were assessed for the model assumptions of normal distribution and constant variance of the residuals. One sheep fed the Hemp diet was not included in the analysis for water intake due to their highly variable intake over the course of the digestibility study.

Nutrient intake, apparent nutrient digestibility, water intake, and nitrogen balance were analyzed using Repeated Measures Analysis, using the Mixed Model procedure in SAS statistical program (SAS Institute Inc., 1997). The analysis examined the fixed effects of “treatment diet” and “day” and the interaction between the fixed effects with “pen” as a random effect and initial liveweight as a covariate. The most appropriate covariance structure for each analysis was determined by referring to the Bayesian Information Criterion (BIC) (Littell et al., 2000). Differences in rumen pH, ruminal ammonia, and VFA concentrations and proportions were analyzed using the Mixed Model procedure in SAS and examined the fixed effect of “treatment diet.” All data were recorded as least squares means ± SE of the least squares means, and any differences were significant when *P* < 0.05.

## RESULTS

### Feed Intake

The average daily intakes of DM (*P* = 0.627), OM (*P* = 0.440), and CP (*P* = 0.175) over the 7 d digestibility study period did not differ between the two diets; however, the NDF (*P* = 0.004) and ADF (*P* = 0.018) intake for Hemp was significantly lower compared with Control (Table 4). On a liveweight basis, the DM intake by the sheep was 3.8 ± 0.15% for Control and 3.6 ± 0.21% for Hemp, although this difference was not significant (*P* = 0.574). For the sheep-fed Hemp, the average daily total THC intake (DM basis) was 4.7 ± 0.31 mg/kg body weight.

**Table 4.** Nutrient (DM basis) intakes over a 7-d period of Merino ewes fed pelleted diets containing either 0% (control) or 42% hemp biomass (hemp)

**Parameter, g/d**	**Diet** ^a^		***P* value**
	**Control**	**Hemp**	
Dry matter	1712.0 (± 79.55)	1655.4 (± 79.54)	0.627
Organic matter	1604.3 (± 73.59)	1520.1 (± 73.59)	0.440
Crude protein	193.5 (± 8.64)	175.5 (± 8.64)	0.175
NDF	650.6 (± 26.70)	504.9 (± 26.70)	0.004
ADF	364.7 (± 15.45)	301.3 (± 15.45)	0.018

^a^Values are least squares means ± standard errors of the least squares means.

NDF, neutral detergent fiber; ADF, acid detergent fiber.

### Water Intake

Sheep-fed Control had a significantly greater (*P* = 0.023) water intake and fecal water output (*P* < 0.001; Table 5). Urinary output volume (*P* = 0.133) and overall water balance (*P* = 0.255); however, were not significantly affected by diet (Table 5).

**Table 5.** Water intake and excretion over a 7 d period of Merino ewes fed pelleted diets containing either 0% (control) or 42% hemp biomass (hemp)

**Parameter, g/d**	**Diet** ^a^		***P* value**
	**Control**	**Hemp**	
Water intake	6494.5 (± 464.79)	4610.5 (± 509.15)	0.023
Urinary output	2244.6 (± 395.68)	1275.8 (± 433.45)	0.133
Fecal water	1582.9 (± 89.92)	926.3 (± 98.50)	<0.001
Water Balance	2667.2 (± 142.39)	2408.1 (± 155.98)	0.255

^a^Values are least squares means ± standard errors of the least squares means.

### Apparent Nutrient Digestibility

As shown in Table 6, there were no differences in the apparent digestibility of DM (*P* = 0.865), OM (*P* = 0.052) and CP (*P* = 0.689) between Control and Hemp. NDF (*P* = < 0.001) and ADF (*P* < 0.001) apparent digestibility were significantly lower for Hemp.

**Table 6.** Apparent nutrient digestibility in Merino ewes fed pelleted diets containing either 0% (control) or 42% hemp biomass (hemp)

**Apparent digestibility, %**	**Diet** ^a^		***P* value**
	**Control**	Hemp	
Dry matter (DMD)	65.3 (± 0.99)	65.6 (± 0.99)	0.865
Organic matter (OMD)	68.1 (± 0.87)	70.8 (± 0.87)	0.052
Crude protein (CPD)	66.4 (± 1.63)	67.3 (± 1.63)	0.689
NDF	42.4 (± 1.91)	27.5 (± 1.91)	<0.001
ADF	41.0 (± 2.21)	18.9 (± 2.21)	<0.001

^a^Values are least squares means ± SEs of the least squares means.

NDF: neutral detergent fiber; ADF: acid detergent fiber.

### Nitrogen Balance

Nitrogen balance (*P* = 0.695), N intake (*P* = 0.175), and fecal N output (*P* = 0.253) were not affected by diet. Urinary N output; however, was significantly lower (*P* < 0.001) in the sheep receiving Hemp (Table 7).

**Table 7.** Daily nitrogen (N) intake, N excretion and N balance of Merino ewes fed pelleted diets containing either 0% (control) or 42% hemp biomass (hemp)

**Parameter, g/d**	**Diet** ^a^		***p* value**
	**Control**	**Hemp**	
Nitrogen intake	31.0 (± 1.38)	28.1 (± 1.38)	0.175
Fecal N output	10.2 (± 0.66)	9.0 (± 0.66)	0.253
Urinary N output	1.9 (± 0.11)	0.7 (± 0.11)	<0.001
N balance	18.9 (± 0.95)	18.3 (± 0.95)	0.695

^a^Values are least squares means ± standard errors of the least squares means.

N: nitrogen.

### Rumen Parameters

Rumen pH (*P* = 0.256) and total VFA concentrations (*P* = 0.713) did not differ between the two diets; however, average ruminal NH_3_ concentration was significantly greater (*P* = 0.024) in those sheep-fed Control (Table 8). The molar proportions of butyric (*P* = 0.039) and hexanoic (*P* = 0.012) acids were significantly greater while the molar proportion of propionic acid was significantly lower (*P* = 0.003) in the sheep-fed Hemp. The molar proportions of acetic (*P* = 0.112), iso-butyric (*P* = 0.616), valeric (*P* = 0.181), iso-valeric (*P* = 0.996), and heptanoic (*P* = 0.317) acids did not differ between diets. The acetic:propionic ratio was significantly greater (*P* = 0.031) while the Pr:Ac 2 × Bu ratio was significantly lower (*P* = 0.003) for Hemp.

**Table 8.** Mean ruminal pH, ammonia-N (mg/L), total volatile fatty acid (VFA) concentration (mmol/L), and individual VFA molar proportions (%) when Merino ewes were fed pelleted diets containing either 0% (control) or 42% hemp biomass (hemp)

**Parameter**	**Diet** ^a^		***P* value**
	**Control**	**Hemp**	
Rumen pH	6.21 (± 0.102)	6.39 (± 0.102)	0.256
Ammonia-N	255.00 (± 25.766)	158.33 (± 25.766)	0.024
Total VFA	88.43 (± 8.315)	92.89 (± 8.315)	0.713
Acetic acid	55.97 (± 1.384)	59.38 (± 1.384)	0.112
Propionic acid	25.99 (± 1.598)	17.37 (± 1.598)	0.003
Butyric acid	15.63 (± 1.400)	20.32 (± 1.400)	0.039
Iso-butyric acid	0.63 (± 0.078)	0.57 (± 0.078)	0.616
Valeric acid	1.37 (± 0.152)	1.68 (± 0.152)	0.181
Iso-valeric acid	0.31 (± 0.072)	0.31 (± 0.072)	0.996
Hexanoic acid	0.08 (± 0.0563)	0.32 (± 0.0563)	0.012
Heptanoic acid	0.01 (± 0.018)	0.04 (± 0.018)	0.317
Pr:Ac 2 x Bu	0.30 (± 0.023)	0.18 (± 0.023)	0.003
Acetic:propionic	2.20 (± 0.414)	3.67 (± 0.414)	0.031

^a^Values are least squares means ± standard errors of the least squares means.

Pr, propionic acid; Ac, acetic acid; Bu, butyric acid.

### Cannabinoid Profiles of Plasma, Ruminal Fluid, and Subcutaneous Fat

No cannabinoids or metabolites were detected in the plasma, fat and ruminal fluid of the sheep-fed Control. A blood sample was not collected from one of the sheep-fed Hemp. The cannabinoids/metabolites detected in the plasma of the sheep-fed Hemp were CBDA (99.38 ± 40.89 µg/L) and THCA (198.00 ± 65.56 µg/L).

The cannabinoids/metabolites detected in the ruminal fluid of the sheep-fed Hemp were CBDA (1,910.00 ± 370.86 µg/L) and THCA (610.50 ± 145.35 µg/L).

On the final day of feeding (i.e., 22 d of full feeding of the experimental diets) the only cannabinoid detected in the subcutaneous fat was ∆^9^-THC and was detected in four of the six sheep-fed Hemp (142, 341, 639, and 713 µg/kg DM). Thirty-five days after the cessation of feeding the experimental diets, ∆^9^-THC was detected in the subcutaneous fat of all six sheep-fed Hemp (18.4, 28.9, 35.2, 54, 60, and 102 µg/kg DM). After 140 d post-feeding, ∆^9^-THC was detected in the subcutaneous fat (33.3 µg/kg DM) of one of the sheep-fed Hemp.

## DISCUSSION

Green hemp biomass, when incorporated in a pelleted diet had no adverse effects on feed intake, apparent nutrient digestibility, or rumen function. However, feeding green hemp biomass for less than 4 wk resulted in the deposition of ∆^9^-THC in the subcutaneous fat and which was still detectable in one sheep 140 d post-feeding.

### Feed Intake

The DM intake during the digestibility study was not affected when green hemp biomass was incorporated into a pelleted diet; however, intake was only measured for 7 d. Similarly, Krebs et al. (2021) found feed intake (in sheep) was not affected when either 50% or 100% of oaten straw was substituted with hemp stubble in a pelleted diet and fed for 56 d. The two pelleted diets used in the current study were formulated to be isocalorific and isonitrogenous and thus it was expected that on this basis there would be no differences in DM intake even though the ingredient composition of the two diets varied. Studies assessing feed intake in sheep typically have a duration of 42 d or more (Ferrell et al., 1986; Muir et al., 2020). Thus, the conduct of a longer duration feeding study on the effects of green hemp biomass on nutrient intake in sheep is warranted to provide more robust data.

While there were no differences between the diets in terms of DM, CP, and OM intakes (Table 4), the sheep-fed Hemp had a significantly lower intake of both NDF and ADF. This lower NDF and ADF intake was not detrimental to the animals and may instead have contributed to increasing total digestible nutrients (Kleinhenz et al., 2020a).

Polyphenolic compounds include proanthocyanidins (Cerrato et al., 2020) and when included at greater levels in ruminant diets, proanthocyanidins can lead to a decrease in feed intake (Papachristou et al., 2003; Salem et al., 2006). The polyphenolic content was greater in Hemp (3,130 mg/kg DM) compared with Control containing barley straw (1,890 mg/kg DM); however, given the lack of difference in feed intake, the polyphenolic compounds in green hemp biomass had no adverse effects.

### Water Intake

Water intake was significantly lower for the sheep-fed Hemp, despite there being no differences in DM intake (Table 4) and the same DM content for both pelleted diets (Table 2). The NDF and ADF intakes were significantly lower for sheep consuming Hemp (Table 4), and this may have resulted in decreased chewing and consequently, decreased salivation, requiring the animal to consume less water (Mertens, 1997; Salem et al., 2013).

Water intake is impacted by the energy source of the diet with those greater in fats or starch requiring the animal to consume less water (Macias Franco et al., 2021). Although the analyzed ME and fat contents of the two diets were similar (Table 2), to make the diets isocalorific and isonitrogenous, more canola oil was added to Hemp (1.751%) compared with Control (0.625%; Table 1). The Hemp diet also contained a greater proportion of cereal grains, resulting in a greater starch content (Table 2). Combined, the differences in oil and starch levels may have contributed at least partially to the differences in water intake of the sheep. Other compounds may have been present in the green hemp biomass that alter water use efficiency, warranting further research.

### Apparent Nutrient Digestibility

Apparent digestibility of DM, CP, OM, NDF, and ADF was increased when sheep were fed hemp stubble replacing 50% and 100% of oaten straw in a pelleted diet compared with a control diet (Krebs et al., 2021). In contrast, in the current study, the apparent digestibility of DM, CP, and OM was not affected, and the apparent digestibility of both NDF and ADF was decreased when green hemp biomass was included at 42% in the pelleted diet. The fraction of fiber in the feed has the greatest influence upon digestibility (Jung and Allen, 1995). The NDF and ADF contents of Control were greater, and the sheep-fed Control thus had a significantly greater intake of both NDF and ADF. Increased NDF intake has been associated with decreased digestibility (Mertens, 1997); although, this was not the case for the current study, with no effect on the apparent digestibility of DM and the greater intake of NDF associated with a greater NDF digestibility for Control.

Green hemp biomass contains many plant secondary metabolites (PSM) (Pellati et al., 2018; Cerrato et al., 2020; Nørskov et al., 2021). Many studies refer to the adverse effects of PSM on digestibility (Robbins et al., 1987; Salem et al., 2006; Villalba et al., 2006). Tannins can reduce fiber digestion by directly inhibiting cellulolytic microorganisms or by complexing with lignocellulose and preventing microbial digestion or both (McSweeney et al., 2001). The methane mitigating effects of polyphenolic compounds have been attributed to decreases in fiber digestion (as reviewed by Vasta et al., 2019) and; therefore, it is possible that some of the PSM present in the green hemp biomass may have contributed to the decrease in fiber digestion.

As previously discussed, Hemp contained a greater proportion of cereal grains, and thus had a greater starch content (Table 2). Previous studies have found a positive correlation between starch content and apparent OM digestibility (Abreu and Bruno-Soares, 1998). The composition of the diet, especially in regard to starch, may also have affected NDF digestibility, with NDF digestibility lower for high-starch compared to low-starch diets (van Vuuren et al., 2010).

There was no difference in apparent CP digestibility between the two experimental diets. In contrast, Krebs et al. (2021) reported increased CP digestibility when sheep were fed pelleted diets containing up to 56% hemp stubble; however, in their study the experimental diets were not isonitrogenous, and the greater CP content of the hemp-containing diets was likely the reason for the greater apparent CP digestibility (Colmenero and Broderick, 2006).

### Rumen pH

The rumen pH was not altered by diet and was below the normal range for ruminants fed a roughage-based diet (van Houtert, 1993). This was expected given the inclusion of concentrates in the diets (Sato, 2016). Although the rumen pH for both diets was less than that typical of roughage diets, the rumen pH was still greater than pH 6.1 and thus, there was a decreased likelihood of any adverse effects on cellulolysis (Mould et al., 1983). This pH would allow optimal microbial protein synthesis as the pH did not fall below 5.7 (van Houtert, 1993) for either diet; however, the ruminal fluid sample was only taken 3 h after initial feeding. Assessing diurnal variations in rumen pH (using rumen-fistulated animals) would provide a greater understanding of the effects of feeding green hemp biomass on rumen pH (as well as ruminal NH_3_ and VFA concentrations).

### Ruminal Ammonia Concentrations

For both diets, the average ruminal NH_3_ concentration was above the minimum threshold of 50–80 mg/L (Satter and Slyter, 1974), indicating that microbial protein synthesis and fermentation of carbohydrates were not hindered. However, as previously discussed, only one ruminal fluid sample was collected; therefore, it cannot be determined if diurnally there were periods when ruminal ammonia concentrations were below the minimum threshold for microbial protein synthesis.

Ruminal NH_3_ concentration was significantly lower when the sheep were fed Hemp, despite there being no differences in dietary CP intake (Table 4), indicating the greater polyphenolics (Table 2) content of Hemp may have increased rumen undegradable protein (RUP), but not indigestible CP as there were no differences in apparent CP digestibility between the two diets (Table 6). Proanthocyanidins bind to dietary proteins (Robbins et al., 1987; Salem et al., 2006), creating a proanthocyanidin–protein complex which is very resistant to fermentation by ruminal microbes (Tanner et al., 1994), resulting in RUP.

Ruminal NH_3_ concentrations did not differ when sheep were fed pelleted diets containing up to 56% hemp stubble (Krebs et al., 2021). Polyphenol concentrations decrease as *Cannabis* plants mature (André et al., 2020; Tremlová et al., 2021), and this would decrease the extent of the formation of proanthocyanidin-protein complexes and the subsequent effect on ruminal NH_3_ concentrations.

Therefore, including green hemp biomass in ruminant diets had positive effects on N utilization. The formation of proanthocyanidin-protein complex in the rumen could explain the decreases in ruminal NH_3_ concentrations (but not below the minimum threshold for microbial activity).

### Nitrogen Balance

The N balance of an animal reflects the gain or loss of total body protein based on the intake and excretion of N. Negative effects on rumen fermentation and microbial protein synthesis occur when N balance falls below −0.3 g/MJ ME (Lebzien et al., 2006). The diet fed to the sheep had no effect on N balance, with all sheep being in positive N balance (Table 7).

There were no differences in N intake and fecal N output between the two dietary treatments; however, urinary N output was significantly lower in the sheep-fed Hemp compared with those fed Control. Excess ruminal NH_3_ not utilized for microbial protein synthesis is transferred via the blood to the liver, where it undergoes detoxification, with a significant portion lost through urinary excretion (Hristov et al., 2004). The sheep-fed Control had greater ruminal NH_3_ and greater urinary N, indicating a potentially greater wastage of N via urinary excretion (Ludden et al., 2000).

The presence of PSM metabolites, particularly proanthocyanidins, in the green hemp biomass could account for both the lower ruminal NH_3_ concentrations (Waghorn et al., 1994), as well as the lower percentage of feed N lost to urine (Carulla et al., 2005; Mahgoub et al., 2008; Grainger et al., 2009). The decrease in N loss via urine and ultimately an increased post-ruminal supply of metabolizable protein would result in improved efficiency of N utilization (Stevens et al., 2021).

### Volatile Fatty Acids

As was found with hemp stubble (Krebs et al., 2021), green hemp biomass had varying effects on the molar proportions of VFA. The molar proportion of butyric acid was greater, while that of propionic acid was lower in the sheep-fed Hemp. Consequently, the ratio of acetic: propionic acid was greater and the ratio of Pr:Ac 2 × Bu was lower for Hemp, indicating that less energy was available to the animals due to less moles of ATP produced and larger energy losses via CH_4_ emissions (McGinn et al., 2004). These effects upon these major VFA were not observed when sheep were fed pelleted diets containing hemp stubble (Krebs et al., 2021).

Flavonoids, as present in hemp, may reduce the proportions of acetic acid and propionic acid, leading to an increase in butyric acid (Oskoueian et al., 2013); although, increased proportions of propionic acid have been previously reported (Seradj et al., 2014). In the current study, it appears flavonoids did not influence total VFA concentrations (as they did not differ between the diets) but may have contributed to the increase in the molar proportions of butyric acid.

It is likely that the analysis of a single ruminal fluid sample together with differences in the composition of the experimental diets contributed to the observed differences in the major VFA. Results from an *in vitro* study showed that barley grain resulted in a lower Pr:Ac 2 × Bu ratio compared with maize grain (Tóthi et al., 2009). Further, rumen fermentation possesses circadian patterns in pH and VFA concentrations, and due to barley being highly degradable in the rumen (Nikkhah, 2012), it is likely that the sheep-fed Hemp had an earlier fermentation peak than those fed Control. Thus, the results of this study may not be representative of the effects of hemp on average (diurnal) concentrations and molar proportions of the major VFA. Having a similar composition for the two diets would also have made interpretation of the VFA results easier, but without the variation in ingredients it was not possible to formulate isocalorific and isonitrogenous diets, given the significant differences in the nutritive value of barley straw (used in Control) and the green hemp biomass (Table 1).

The molar proportions of all minor VFA were significantly increased when hemp stubble was included in a pelleted diet (Krebs et al., 2021) whereas inclusion of green hemp biomass increased only the molar proportion of hexanoic acid and had no effect on the molar proportions of iso-butyric, valeric, iso-valeric or heptanoic acids. Hexanoic acid production has been identified to increase when pH is closer to neutral (Kenealy et al., 1995), with the pH of the sheep-fed Hemp diet tending to be closer to neutral. In cattle, greater concentrations of hexanoic acid coincided with increased concentrations of butyric acid when fed purified diets (Orskov et al., 1967), which aligns with this study. Further research is required to better understand the effects of green hemp biomass upon VFA and consequently, energy metabolism and ruminant production.

### Cannabinoids

Both CBDA and THCA were detected in the plasma of the sheep-fed Hemp. Only THCA was detectable in the plasma when sheep were fed hemp stubble and at much lower concentrations (10–11 µg/L) (Krebs et al., 2021) than in the current study (99.38 μg/L). The greater concentration of THCA detected in the plasma could be due to increased THCA of green hemp biomass (0.014% w/w) compared to hemp stubble (0.002% w/w). Similarly, the detection of CBDA in the current study would be associated with the increased concentrations in the pelleted diet based on green hemp biomass (0.041% w/w) compared to hemp stubble (<0.001% w/w) (Krebs et al., 2021). Overall greater content of cannabinoids for green hemp biomass compared to hemp stubble was expected as the stem contains only trace amounts of cannabinoids (Cappelletto et al., 2001), and the green hemp biomass used in this study was harvested around flowering, which is the growth stage where cannabinoid concentrations peak in the plant (Burgel et al., 2020).

Cattle orally dosed with industrial hemp (5.4 mg of CBDA/kg BW) had detectable concentrations of THCA, CBDA, CBDVA, and CBCA in the plasma (Kleinhenz et al., 2020b). Neither CBDVA nor CBCA were analyzed in the plasma of the sheep-fed Hemp in the current study nor in sheep-fed diets containing hemp stubble (Krebs et al., 2021). Further research is required to determine the pharmacokinetics of cannabinoids of green hemp biomass in ruminant animals, especially to gain an understanding of the half-life and the rate of elimination for the detectable cannabinoids.

Both CBDA and THCA were detected in the ruminal fluid of all animals fed Hemp. In fiber-type hemps CBDA is the cannabinoid found in the greatest concentration followed by THCA (Andre et al., 2016). In cattle, it was found that CBDA was slowly but readily absorbed by the rumen after a single oral dose of industrial hemp (Kleinhenz et al., 2020b), providing a long duration for detection in ruminal fluid. The concentrations of CBDA and THCA in the Hemp diet utilized in the current study were 0.041% and 0.014%, respectively (Table 2). All other cannabinoids in the Hemp diet were <0.001% (Table 2) contributing to no other cannabinoids being of a detectable concentration in the ruminal fluid.

At the end of the feeding trial, ∆^9^-THC was detected in the subcutaneous fat of four of the six sheep-fed Hemp. Krebs et al. (2021) also reported Δ^9^-THC (range 76–290 µg/kg) in the subcutaneous fat of sheep-fed diets containing hemp stubble. Despite the longer period of feeding the hemp-stubble-containing diets (56 d) compared to only 22 d for the current study, subcutaneous fat cannabinoid residues were generally lower when sheep were fed the hemp stubble. Differences in the total THC concentrations of the hemp stubble diet (0.002%) compared to green hemp biomass diet (0.015%) likely account for the differences in residue concentrations between studies.

Under normal conditions, ∆^9^-THC passively diffuses from fat deposits back into the blood, contributing to its long elimination half-life (Gunasekaran et al., 2009). All of the sheep had detectable levels of ∆^9^-THC in subcutaneous fat 35 d post-feeding, with residues still remaining in one sheep 140 d post-feeding, supporting the slow elimination of ∆^9^-THC from subcutaneous fat (Huestis, 2007). The increase in the number of sheep with detectable levels of ∆^9^-THC 35 d post-feeding (compared to the end of the feeding trial) supports the redistribution of ∆^9^-THC from other tissues in the body to the subcutaneous fat (Karschner et al., 2012).

Currently, in Australia, there is zero tolerance for THC in foods of animal origin (meat, milk, eggs) until FSANZ sets a safe or “maximum level” (FSANZ, 2012). Given that ∆^9^-THC could still be detected in the subcutaneous fat of one sheep 140 d post-feeding indicates that establishment of a withholding period may not be practical for animals destined for market within 6 mo, but may be suitable for other livestock categories such as wool-producing sheep or pregnant/lactating ewes.

## Conclusion

Green hemp biomass at the flowering stage seems to be a suitable forage for ruminants in terms of feeding value. However, cannabinoid detection in subcutaneous fat means management strategies will need to be implemented to ensure no detectable cannabinoids are present in marketable goods. Alternatively, a maximum limit for cannabinoids in animal products should be set.
